# Establishment of human distal lung organoids for SARS-CoV-2 infection

**DOI:** 10.1038/s41421-021-00346-2

**Published:** 2021-11-09

**Authors:** Ting Wang, Ning Zhang, Shipan Fan, Lianzheng Zhao, Wanlu Song, Yuhuan Gong, Quan Shen, Cheng Zhang, Peng Ren, Chutong Lin, Wei Fu, George F. Gao, Shaohua Ma, Yuhai Bi, Ye-Guang Chen

**Affiliations:** 1 The State Key Laboratory of Membrane Biology, Tsinghua-Peking Center for Life Sciences, School of Life Sciences, Tsinghua University, Beijing, China; 2 CAS Key Laboratory of Pathogenic Microbiology and Immunology, Institute of Microbiology, Center for Influenza Research and Early-Warning, CAS-TWAS Center of Excellence for Emerging Infectious Diseases, Chinese Academy of Sciences, Beijing, China; 3 Max-Planck Center for Tissue Stem Cell Research and Regenerative Medicine, Guangzhou Regenerative Medicine and Health Guangdong Laboratory, Guangzhou, China; 4 Department of Thoracic Surgery, Peking University Third Hospital, Beijing, China; 5 Department of General Surgery, Peking University Third Hospital, Beijing, China; 6 University of Chinese Academy of Sciences, Beijing, China

**Keywords:** Adult stem cells, Cell growth

Dear Editor,

COVID-19 caused by severe acute respiratory syndrome coronavirus 2 (SARS-CoV-2) is being a serious pandemic with more than 164 million infections and 3.41 million deaths in over 200 countries as of 20 May 2021. This deadly disease mainly affects the respiratory system, gastrointestinal tract, and nervous system. To understand the mechanisms underlying SARS-CoV-2 infection and develop effective medicines, appropriate models that can be used to faithfully mimic viral infection in the human body are urgently needed. Several cell lines have been commonly used to investigate infection susceptibilities, virus infection, replication mechanism and to screen antiviral drugs^1,2^. Mouse models expressing human ACE2 and hamsters have also been used to imitate the SARS-CoV-2 infection^3^. However, both cell lines and animal models have limitations and cannot accurately capture the key characteristics of human biology. As a new type of research model, human pluripotent stem cells (hPSCs)-derived organoids, like lung, colon, brain have been used for SARS-CoV-2 infection^4,5^. However, these hPSC-derived organoids represent a fetal phenotype but not a fully mature state in adults. Human lung alveolar type 2 cells-based 3D cultures, human 2D air–liquid interface bronchioalveolar and human small intestinal organoid models were also used^6,7^. Here, we established human distal lung organoids (hDLO) from distal lung parenchymal tissues to investigate the infection dynamics of SARS-CoV-2, and observed cellular dynamic changes in the infected organoids, which are similar to clinical features in COVID-19 patients.

Human DLOs were established from surgically fresh distal lung parenchymal tissues (see Supplementary Data). After cultured in chemically defined medium, the organoids appeared round with obvious lumen (Fig. 1a). These organoids exhibited bronchial structures with rocking cilia driving mucus movement (Supplementary Video S1). To clarify the cell types in the organoid system, qPCR analysis of cell marker expression was performed with hDLOs and their paired tissue. The result revealed that the major cell markers were expressed in the organoids, including basal cells (KRT5, PROM1), goblet cells (MUC20, MUC5AC), ciliated cells (TUBA1A, FOXJ1), club cells (SCGB1A1, KLF5), AT2 cells (SFTPD, DCLAMP), and AT1 cells (AQP5, PDPN) (Supplementary Fig. S1a), which was confirmed by immunofluorescence analysis of hDLOs that showed six cell types, including airway cells in lung bronchia: KRT5^+^ basal cells, ACCTUB^+^ ciliated cells, MUC5AC^+^ goblet cells, CC10^+^ club cells, and alveolar cells in alveoli: AQP5^+^ AT1 cells, SFTPB^+^ AT2 cells (Fig. 1b). In addition, we also observed that the AT2 cell marker SFTPC was co-expressed with the basal cell marker KRT5 or the ciliated cell marker ACCTUB in single organoid (Supplementary Fig. S1b). Similarly, the AT1 cell marker PDPN was co-expressed with the club cell marker CC10 or the goblet cell marker MUC5AC, and the AT1 cell marker AQP5 was co-expressed with the ciliated cell marker ACCTUB. Thus, the organoid in our culture is a mixture of multiple cell types, including alveolar cells and airway cells.

**Fig. 1:**
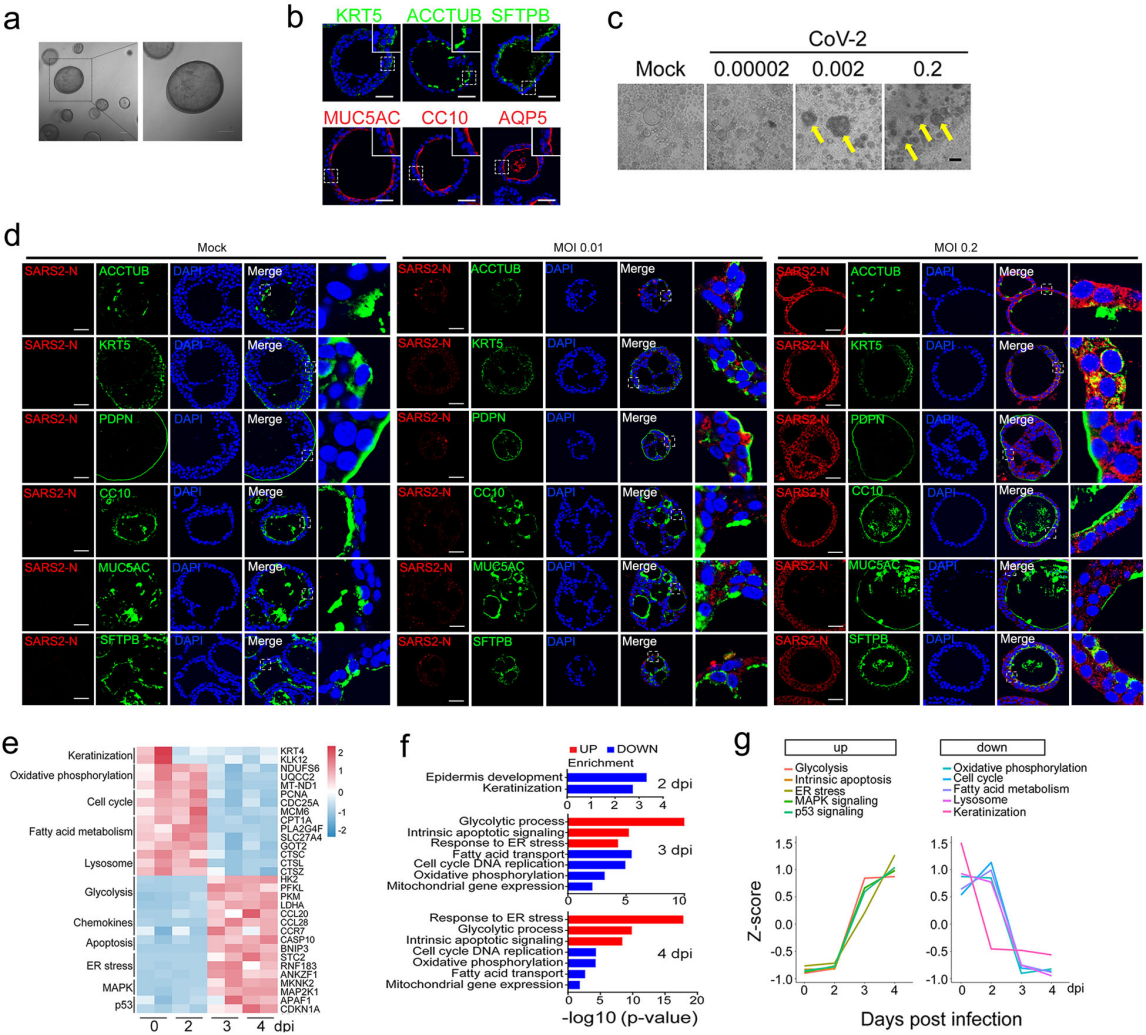
SARS-CoV-2 infection of human distal lung organoids and its serial cellular responses. **a** Representative microscopy images of tissue-derived hDLOs. Scale bar, 100 μm. **b** Immunofluorescence staining of different cell markers in hDLOs. Scale bar, 50 μm. **c** Representative images of hDLOs upon SARS-CoV-2 infection at 3 dpi with indicated MOIs. Yellow arrows indicate the cytopathic organoids caused by SARS-CoV-2. Scale bar, 100 μm. **d** Immunofluorescence staining of different cell markers and SARS-CoV-2-N antibodies in mock or SARS-CoV-2-infected hDLOs at 3 dpi at MOI = 0.01 and 0.2. Scale bar, 50 μm. The data were repeated for three times. **e** Heatmap of differentially expressed genes in SARS-CoV-2-infected hDLOs compared to mock infection. **f** GO analysis of differentially expressed genes in SARS-CoV-2-infected hDLOs at 2, 3, and 4 dpi. The *x*-axis represents −log10 (*P* value) for the enrichment analysis. **g** Variation trends of main pathways along different time points were shown.

Then, hDLOs were infected with SARS-CoV-2 at an increasing concentration of 0.00002, 0.002, 0.2 MOI (multiplicity of infection), respectively. At 3 days post-infection (dpi), the organoids exhibited cytopathic effects such as losing cellular structures and being winkled with 0.002 MOI virus, and the effects were more obvious with 0.2 MOI virus (Fig. 1c), indicating that the organoids were readily infected. Viral infection was confirmed by the expression of viral N RNA, S RNA, and N protein in the infected hDLOs (Supplementary Fig. S1c, d).

SARS-CoV-2 was found in ciliated cells, club cells, goblet cells, AT1, and AT2 cells of COVID-19 autopsy lungs and bronchial epithelium^8,9^. It had been reported that the virus could enter into different cell types: ciliated, club, goblet, and AT2 cells^4,6,7,10^. In order to clarify which cell type was permissive to SARS-CoV-2 entry in hDLOs, immunostaining was performed. As shown in Supplementary Fig. S1e, SARS-CoV-2 N protein was detected in all the cells, indicating that all the cell types can be infected. Indeed, co-staining revealed that N protein was detected in ACCTUB^+^ ciliated cells, KRT5^+^ basal cells, CC10^+^ club cells, MUC5AC^+^ goblet cells, PDPN^+^ AT1 cells, SFTPB^+^ AT2 cells in hDLOs. Moreover, immunostaining of SARS-CoV-2 at low MOI condition (MOI = 0.01) was observed in five cell types except ciliated cells (Fig. 1d). This is consistent with our immunostaining data that both the viral receptors ACE2 and TMPRSS2 were expressed in all six cell types (Supplementary Fig. S2), which was reported by single-cell RNA sequencing data^11,12^. Although various cell types have been reported to be permissive to SARS-CoV-2 infection in different models, none showed that all the six cell types were infected in a single study. The reason could be that the organoids we employed were directly derived from adult lung tissues and had a better preservation of mature cell types.

Hematoxylin and eosin (H&E) staining showed that SARS-CoV-2 infection caused a looser structure of hDLOs (Supplementary Fig. S3a), and reduced mucin expression as detected by Periodic Acid Schiff staining (Supplementary Fig. S3b), which is in line with the results observed in the infected peripheral lung^8^. Co-immunostaining showed that except ciliated cells, other five cell types were positive for Ki67 staining in SARS-CoV-2-infected organoids (Supplementary Fig. S3c), suggesting they were proliferating cells in lung organoids after infection.

To exploit the cellular responses upon SARS-CoV-2 infection, transcriptomic profiling was examined following viral infection at 2, 3, and 4 dpi. Volcano plots at different time points showed significant enrichment of SARS-CoV-2 sequence at 2 dpi, reaching the maximum at 3 dpi (Supplementary Fig. S4a). Virus infection caused a dramatic change of gene expression: 9 genes were significantly downregulated and 34 genes were upregulated at 2 dpi; 564 downregulated and 783 upregulated at 3 dpi; 1007 downregulated and 1039 upregulated at 4 dpi (Supplementary Table S1). Among the downregulated genes at 2 dpi, keratinization-related KRT4 and KLK12 were enriched as determined by Gene Ontology (GO) analysis, which is consistent with previous reports^6^.

SARS-CoV-2 infection caused a cellular metabolic switch from oxidative phosphorylation to glycolysis at 3 and 4 dpi (Fig. 1e, f). The expression of glycolysis genes such as hexokinase 2 (HK2), phosphofructokinase (PFKL), pyruvate kinase M1/2 (PKM), and lactic dehydrogenase (LDHA) was increased, while that of the genes involved in mitochondrial respiratory chain complexes, such as NADH: ubiquinone oxidoreductase subunit S6 (NDUFS6), ubiquinol-cytochrome C reductase complex assembly factor 2 (UQCC2) and mitochondrial genes mitochondrially encoded NADH: ubiquinone oxidoreductase core subunit 1 (MT-ND1) and 2 (MT-ND2) was reduced (Fig. 1e and Supplementary Table S2). qRT-PCR confirmed some of the results (Supplementary Fig. S4c). GO and KEGG analyses also confirmed the upregulation of glycolysis and downregulation of oxidative phosphorylation (Fig. 1f and Supplementary Fig. S4b, Tables S3 and S4). Our results are consistent with recent reports that glycolysis was enhanced upon SARS-CoV-2 infection, and aerobic glycolysis might be necessary for viral replication^1^. The inhibition of oxidative phosphorylation and mitochondrial dysfunction was also observed in COVID-19 sepsis^13^.

SARS-CoV-2 hijacks the endoplasmic reticulum (ER) membrane for replication and provoked ER stress^14^. Indeed, we observed that ER stress was activated in the infected hDLOs (Fig. 1e, f). Viral infection also reduced DNA replication and fatty acid metabolism. Moreover, we observed a downregulation of lysosome genes in hDLOs at 4 dpi (Fig. 1e and Supplementary Fig. S4b), which was proposed to be important for β-coronaviruses egress^15^.

SARS-CoV-2 infection induced a cascade of reactions leading to intrinsic apoptosis and cell cycle arrest (Fig. 1e, f and Supplementary Fig. S4b). Apart from increased caspases and other apoptotic genes at 3 dpi, chemokines such as CCL5, CCL20, and CCL28 were also upregulated, accompanied by activation of MAPK and p53 signaling pathways (Fig. 1e and Supplementary Fig. S4b), which was observed in the phosphorylation landscape irritated by SARS-CoV-2 infection^2^.

Mfuzz cluster analysis of transcriptomic expression patterns at different time points uncovered six clusters of genes representing different expression trends during SARS-CoV-2 infection (Supplementary Fig. S4d and Table S5). Most genes in Cluster 1 and Cluster 2 represented downregulated and upregulated trends at 3 dpi, respectively. Genes involved in oxidative phosphorylation were aggregated in Cluster 1, while genes associated with glycolysis gathered in Cluster 2. In agreement with the above observations, analysis of variation trends of main pathways showed that glycolysis, apoptosis, ER stress, MAPK, and p53 pathways showed an upregulation trend, while oxidative phosphorylation, cell cycle, fatty acid metabolism, lysosome, and keratinization pathways exhibited a downregulation trend (Fig. 1g). Together, based on the characteristic analysis along infection time points, our organoid model could simulate the infection process observed in clinical pathology to some extent.

In summary, we generated hDLOs from distal lung tissues and found that SARS-CoV-2 could infect all these cell types. The infected hDLOs exhibited pathological changes, similar to clinical features in COVID-19 patients. Transcriptomic analysis of the infected hDLOs revealed the serial cellular responses: downregulation of keratinization at the early stage of virus infection, followed by a metabolic switch from oxidative phosphorylation to glycolysis, reduced fatty acid metabolism, ER stress, cell cycle arrest, apoptosis, and decreased lysosomal function. Therefore, the lung organoid model can be used to faithfully mimic the infection process of SARS-CoV-2 and should be a great platform for antiviral drug discovery.

## Supplementary information

Supplementary Video S1

Supplementary Information

## Data Availability

The latest data on the COVID-19 global outbreak is available from https://www.who.int/data#reports.
