# Supplementary material for: Establishment of human distal lung organoids for SARS-CoV-2 infection
Source: Cell Discov. 2021 Nov 9;7:108. doi: 10.1038/s41421-021-00346-2 (PMC8575945; doi:10.1038/s41421-021-00346-2)
Supplement: Supplementary file 2 — Supplementary Information [file 41421_2021_346_MOESM2_ESM.zip › Supplementary Figures and Data_ESM.pdf]

Supplementary information

Establishment of human distal lung organoids for SARS-CoV-2 infection

Ting Wang[1]*, Ning Zhang[2]*, Shipan Fan[3]*, Lianzheng Zhao[1]*, Wanlu Song[1]*, Yuhuan Gong[2], Quan Shen[2], Cheng Zhang[2], Peng Ren[4], Chutong Lin[4], Wei Fu[5], George F. Gao[2], Shaohua Ma[4#], Yuhai Bi[2,6#] and Ye-Guang Chen[1,3#]

Supplementary Methods
Supplementary Figures S1-S4
Supplementary Video S1
Supplementary Tables S1-S6

Supplementary Methods

Human tissue collection and ethics statement

Human distal lung parenchymal tissues, above 5-cm away from the tumor border in lung cancer patients, were freshly processed. All the samples were collected from Peking University Third Hospital (Beijing, China) according to the guidelines of the Declaration of Helsinki and approved by Peking University Third Hospital Medical Science Research Ethics Committee (M2020229).

Human lung distal lung organoids culture

Human lung distal lung organoids were established as previously described [1]. Briefly, human distal lung parenchymal tissues were washed twice in cold D-PBS and minced into small pieces with a scalpel, then transferred to 5 mM EDTA in PBS for 15 min at room temperature, and digested in 1 mM EDTA in TrypLE for 1 h at 37 ^{o}C with agitation. The pieces were collected in cold Advanced DMEM/F12, further dissociated to obtain tissue cell suspension and filtered through a 70 μm cell strainer to remove large debris. Cell pellets were centrifuged at 300g for 5 min after washed with cold D-PBS and resuspended with cold Matrigel. Three drops (10000 cells/drop) of 30ul mixture were plated into one well of a 12-well plate. After drops were solidified at the incubator for 10 min, 1 ml pre-warmed human lung organoid medium (Advanced DMEM/F12(12634028; Thermo Scientific) supplemented with N2 (1750202; Thermo Scientific), B27 (17504044; Thermo Scientific), penicillin/streptomycin (15140122; Thermo Scientific), Antibiotic-Antimycotic (100 U/ml; Thermo Scientific), HEPES (10 mM; Thermo Scientific), GlutaMax (2mM; Thermo Scientific), N-acetyl-L-cysteine (1.25 mM; Sigma-Aldrich) , Primocin (50ug/ml; Invitrogen) containing Noggin (100 ng/ml;

R&D), R-spondin 1 (500 ng/ml; R&D), A83-01 (0.5 μM; Cayman), Y-27632 (5 μM; Selleck), FGF-7 (25 ng/ml; Novoprotein), FGF-10 (100 ng/ml; Novoprotein), SB202190 (5μM; Selleck), Nicotinamide (5 mM; Sigma-Aldrich)) were added. The medium was replaced every 4 days and organoids were passaged at 1:2-1:3 every 2 weeks.

SARS-CoV-2 infection of organoids

SARS-CoV-2 (hCoV-19/China/CAS-B001/2020, National Microbiology Data Center NMDCN0000102-3, GISAID databases EPI_ISL_514256-7) was identified by Dr. Yuhai Bi's team in the Institute of Microbiology, Chinese Academy of Sciences, and propagated in Vero E6 cells. Organoids were plated into a 12-well plate with 100 organoids per well. Organoids were then transferred to the BSL-3 facility and infected with SARS-CoV-2. After three days' infection, organoids were collected and lysed in TRIzol for RNA extraction or fixed by PFA for H&E, Periodic Acid-Schiff assay and immunofluorescence. All infection experiments were conducted in three independent experiments with appropriate controls included.

Viral RNA RT-qPCR

Viral RNA from organoids lysates was extracted using QIAamp Viral RNA Kit (Qiagen) according to the manufacturer's instruction and quantified by COVID-19 virus (2019-nCoV) Triple-Detection Kit (Real-Time PCR Method) (MABSKY BIO-TECH CO., LTD.), which was designed with specific primers and Taqman probes for the conserved regions of the viral S and N gene sequence. The PCR condition was as follows: 50°C for 30 min, 95°C for 3 min, followed by 45 cycles of 95°C for 3 s and 55°C for 30 s. Plasmids containing the complete SARS-CoV-2 S gene and N gene sequence were serially diluted (10-fold) to generate a standard curve for quantification

of viral RNA copies. A pair of primers and probe of Human internal reference gene (RNP) are used as quality control of sampling, RNA extraction and real-time PCR.

Histochemistry

Organoids were fixed with 4% paraformaldehyde at 4 ^{o}C overnight, washed with PBS, then paraffin-embedded and sectioned (5 μm). Sections were deparaffinized and stained with H&E and Periodic Acid-Schiff assay for histological analysis and images were acquired on Digital Pathological Section Scanner (KF-PRO-120, KFBIO).

Immunofluorescence assay

Organoid sections were de-paraffinized in isopropanol and dehydrated by a graded alcohol series, followed by antigen retrieval and permeabilized for 20 min in PBS with 0.2% Triton X-100 for 20 min at 4 ^{o}C. Then the sections were blocked in 1% BSA/PBS (blocking buffer) for 45 min at room temperature before incubated with the primary antibody at 4 ^{o}C overnight. The fluorescein-labeled secondary antibodies (1:300, Life Technologies) were added for 90-120 min at room temperature. Confocal laser scanning (FV3000; Olympus) was used to detect the staining signals. The antibodies for rabbit anti-Muc5ac (1:100, ab198294; Abcam), mouse anti-Muc5ac (1:100, ab3649; Abcam), mouse anti-Acetylated α-tubulin (1:50, sc-23950; Santa Cruz Biotechnology), mouse anti-PDPN (1:50, 67432-1-Ig; Proteintech), rabbit anti-AQP5 (1:100, ab92320; Abcam), rabbit anti-CC10 (1:50, 10490-1-AP; Proteintech), rabbit anti-SFTPC (1:50,10774-1-AP; Proteintech), mouse anti-SFTPB (1:50, sc-133143; Santa Cruz Biotechnology), rabbit anti-Sars-Cov-2 Nucleoprotein (1:100, 40143-R001; Sino Biological), mouse anti-CK5 (1:50, BF0493; Affinity Biosciences), mouse anti E-cadherin (1:300, 610182; BD Biosciences), goat anti-ACE2 (1:100, AF933; R&D

Systems), rabbit anti-TMPRSS2 (1:100, 14437-1-AP; Proteintech), rabbit anti-Ki67 (1:100, ab15580; Abcam), mouse anti-Ki67 (1:100, 9449s; Cell Signaling Technology) were used in immunofluorescence assay.

RNA isolation and quantification

RNA was isolated from cultured organoids and distal lung parenchymal tissues by using TRIzol Reagent (Life Technologies). Total RNA yield was determined by using NanoDrop 2000 (Thermo Fisher Scientific). cDNAs were generated using Revertra Ace (Toyobo). Quantitative real-time PCR (qRT-PCR) were carried out in triplicates on the LightCycler 480 (Roche). Primers used were listed in Supplementary Table S6.

RNA-Sequencing and bioinformatics analysis

Organoids infected with SARS-CoV-2 were performed at MOI=0.1 and cells were collected at indicated time points. Total RNA was extracted using TRIzol (Invitrogen) and RNA-seq libraries of polyadenylated RNA were prepared using NEBNext® Ultra™ RNA Library Prep Kit for Illumina® according to the manufacturer's instruction. cDNA libraries were sequenced using NovaSeq PE250 platform. For viral RNA analysis, sequencing reads were aligned to the SARS-CoV-2 (hCoV-19/China/CAS-B001/2020, National Microbiology Data Center NMDCN0000102-3, GISAID databases EPI_ISL_514256-7) and human genome reference (GRCh38) using STAR [2]. The R package DESeq2 [3] was used to perform differential expression analysis using gene counts data calculated by STAR. Differential expressed genes with Log2 Fold Change absolute value > 1 and P value < 0.01 were used to do the GO (Gene Ontology) and KEGG (Kyoto Encyclopedia of Genes and Genomes) enrichment analysis using R package clusterProfiler [4]. Differentially expression genes were clustered into

significant discrete clusters with relative expression changes using mFuzz [5]. Enrichment analysis of genes with α-core > 0.75 of each cluster was performed by clusterProfiler.

References

1 Sachs, N. *et al.* Long-term expanding human airway organoids for disease modeling. *EMBO J* **38**, e100300, doi:10.15252/embj.2018100300 (2019).

2 Dobin, A. *et al.* STAR: ultrafast universal RNA-seq aligner. *Bioinformatics* **29**, 15-21, doi:10.1093/bioinformatics/bts635 (2013).

3 Love, M. I., Huber, W. & Anders, S. Moderated estimation of fold change and dispersion for RNA-seq data with DESeq2. *Genome Biol* **15**, 550, doi:10.1186/s13059-014-0550-8 (2014).

4 Yu, G., Wang, L. G., Han, Y. & He, Q. Y. clusterProfiler: an R package for comparing biological themes among gene clusters. *OMICS* **16**, 284-287, doi:10.1089/omi.2011.0118 (2012).

5 Kumar, L. & M, E. F. Mfuzz: a software package for soft clustering of microarray data. *Bioinformation* **2**, 5-7, doi:10.6026/97320630002005 (2007).

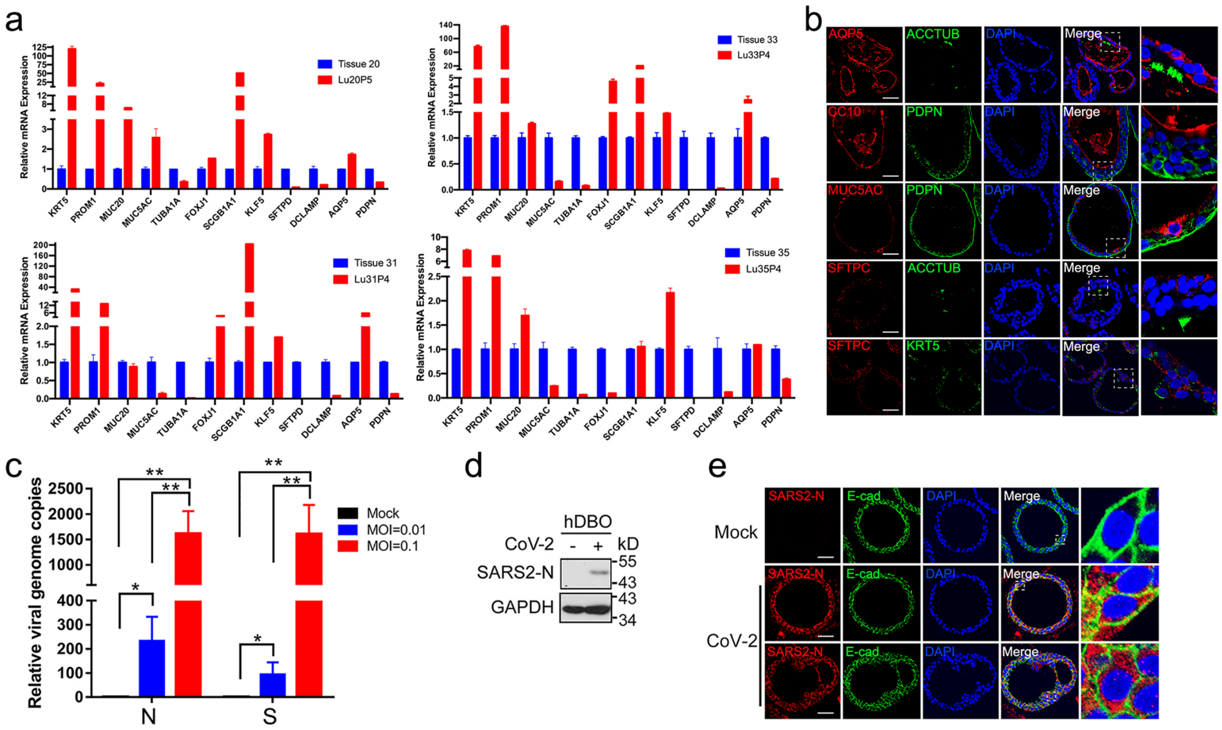

Figure S1. Human distal lung organoids are infected by SARS-CoV-2. (**a**) The mRNA levels of each cell type marker were examined by qRT-PCR in surgical distal lung tissue and paired lung organoids. Data is shown as mean ± SD (n=2). (**b**) Co-immunostaining of airway cell markers and alveolar cell markers in individual organoids. Scale bar = 50μm. (**c**) Relative SARS-CoV-2 viral RNA expression in hDLOs. Viral RNA from infected hDLOs at 3 dpi (MOI = 0.01 or MOI=0.1) was analyzed by qRT-PCR. Statistical analysis was performed by a two-tailed Student's t-test. The data are represented as means ± SD (n = 3). * Indicates $p<0.05$; ** $p<0.01$; *** $p<0.001$. (**d**) Immunoblotting was performed with SARS-CoV-2-N antibody to detect the SARS-CoV-2 infection in hDLOs at 3 dpi (MOI = 1). (**e**) Immunostaining of E-cadherin and SARS-CoV-2-N in mock or SARS-CoV-2-infected hDLOs at 3 dpi (MOI=0.2). Scale bar = 50μm. The data were repeated for three times.

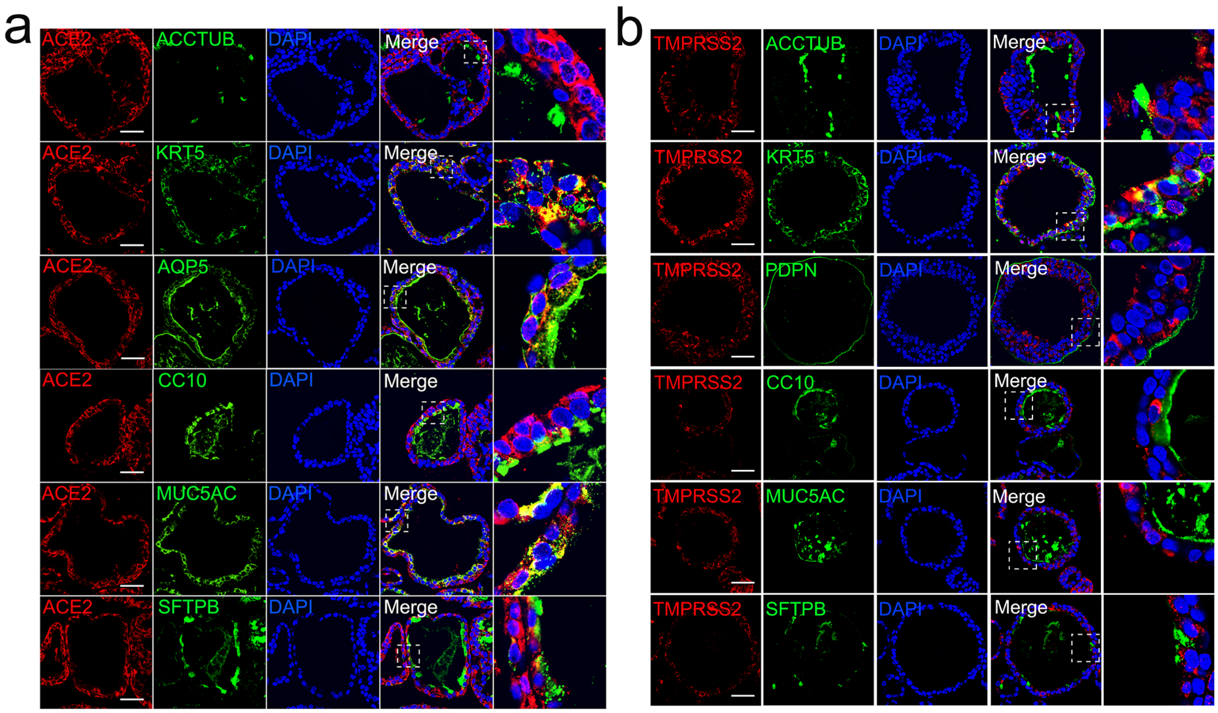

Figure S2. ACE2 and TMPRSS2 were expressed in all six cell types. (**a**) Immunostaining of different cell markers and ACE2 in hDLOs. Scale bar = 50μm. (**b**) Immunostaining of different cell markers and TMPRSS2 in hDLOs. Scale bar = 50μm.

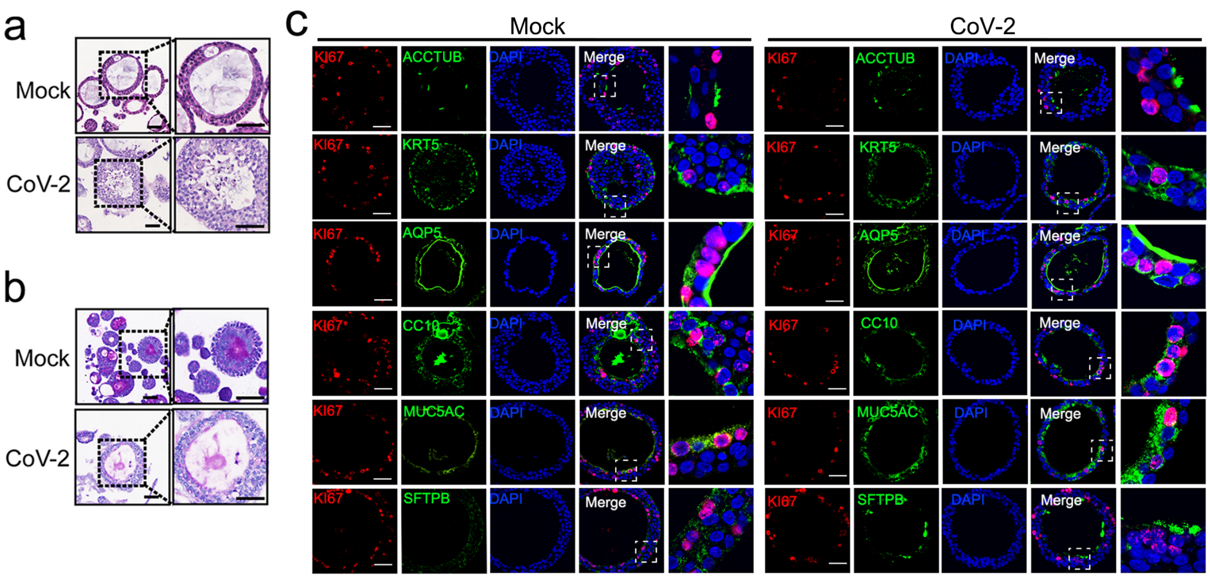

Figure S3. Cellular structure and mucin expression are affected by SARS-CoV-2 infection. (**a**) Representative H&E images of mock or SARS-CoV-2 infected hDLOs at 3 dpi (MOI = 2). Scale bar = 50μm. (**b**) Representative PAS images of mock or SARS-CoV-2 infected hDLOs at 3 dpi (MOI = 2). Scale bar = 50μm. (**c**) Immunostaining of different cell markers and Ki67 in hDLOs. Scale bar = 50μm.

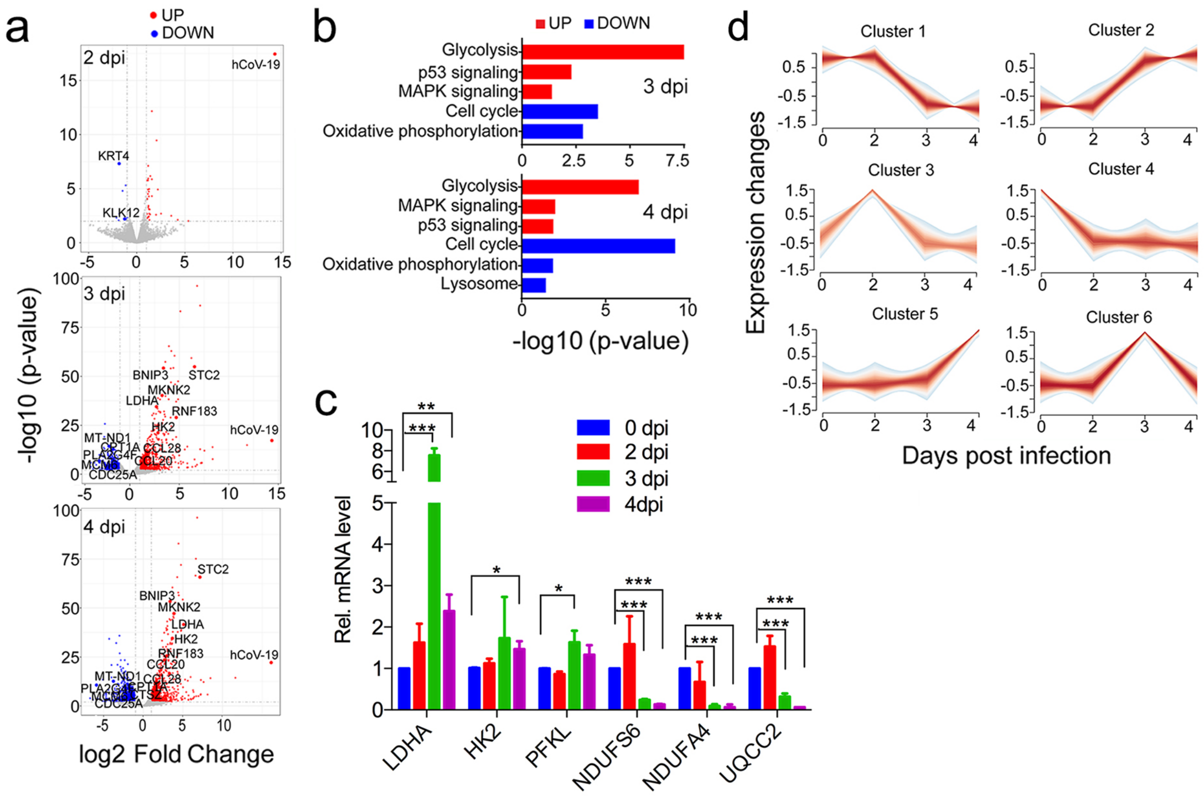

Figure S4. SARS-CoV-2 infection results in serial cellular responses. (**a**) Volcano plots showing differentially expressed genes in mock or SARS-CoV-2-infected hDLOs at 2, 3 and 4 dpi (MOI=0.1). The x-axis represents Log2 Fold Change and y-axis represents -log10 (P value). hCoV-19 was labeled in all conditions. (**b**) KEGG analysis of mock or SARS-CoV-2-infected hDLOs at 2, 3 and 4 dpi. The x-axis represents -log10 (p value) for the enrichment analysis. (**c**) The mRNA levels of metabolic genes were measured by qRT-PCR in SARS-CoV-2 infected organoids at 0 dpi, 2 dpi, 3dpi and 4dpi. The results were collected from three independent experiments. Statistical analysis of the data was performed by two-tailed Student's t-test. * Indicates $p<0.05$; ** $p<0.01$; *** $p<0.001$. (**d**) Genes were clustered with relative expression changes using mFuzz. n=2 biological independent experiments.

Supplementary Video S1. Human lung distal lung organoids.

Supplementary Table S1. Differentiated expression analysis.

Supplementary Table S2. Significant differentiated genes in heatmap.

Supplementary Table S3. GO analysis of mock and SARS-CoV-2 infected hDLOS.

Supplementary Table S4. KEGG analysis of mock and SARS-CoV-2 infected hDLOS.

Supplementary Table S5. Differentiated expression genes in clusters.

Supplementary Table S6. Sequence of the qPCR primer pairs.
